# Half-Heusler (TiZrHf)NiSn Unileg Module with High Powder Density

**DOI:** 10.3390/ma6041326

**Published:** 2013-03-27

**Authors:** Sascha Populoh, Oliver C. Brunko, Krzysztof Gałązka, Wenjie Xie, Anke Weidenkaff

**Affiliations:** Solid State Chemistry and Catalysis Laboratory, Empa—Swiss Federal Laboratories for Materials Testing and Research, Ueberlandstrasse 129, Duebendorf CH-8600, Switzerland; E-Mails: sascha.populoh@empa.ch (S.P.); oliver.brunko@empa.ch (O.C.B.); krzysztof.galazka@empa.ch (K.G.); wenjie.xie@empa.ch (W.X.)

**Keywords:** thermoelectricity, thermoelectric modules, half-Heusler compounds

## Abstract

(TiZrHf)NiSn half-Heusler compounds were prepared by arc melting and their thermoelectric properties characterized in the temperature range between 325 K and 857 K, resulting in a Figure of Merit ZT ≈ 0.45. Furthermore, the prepared samples were used to construct a unileg module. This module was characterized in a homemade thermoelectric module measurement stand and yielded 275 mW/cm^2^ and a maximum volumetric power density of 700 mW/cm^3^. This was reached using normal silver paint as a contacting material; from an improved contacting, much higher power yields are to be expected.

## 1. Introduction

Thermoelectricity (TE) offers the unique opportunity of a direct conversion of waste heat into electricity. Until now, the broader use of this technique is hindered by toxicity and scarcity of the containing elements. Environmentally friendly alternatives are needed with an increased efficiency of thermoelectric conversion. The efficiency of thermoelectric conversion is determined by the dimensionless Figure of Merit ZT = S^2^·σ/κ, where S is the Seebeck coefficient, σ the electrical conductivity and κ the thermal conductivity, which can be described as the sum of a lattice part, κ_l_, and an electronic part, κ_e_:κ = κ_l_ + κ_e_.

One prime example for environmentally benign thermoelectric materials for high temperatures are the complex transition metal oxides, showing, until now, unfortunately, a rather moderate performance. Recently, spin-orbit coupling [1,2] or metal-insulator transitions [3] were discussed as possible ways to enhance the performance, but a ZT = 1, the benchmark for practical use of TE materials, still remains difficult to achieve. 

Another promising group of materials are the cubic half-Heusler (HH) compounds with the chemical formula XYZ. HH compounds with 18 valence electrons are found to be semiconducting and are exhibiting high Seebeck coefficients, high electrical conductivities, but also too high thermal conductivities for a practical application as a thermoelectric material [4]. The XNiSn (with X = Ti, Zr, Hf) family is found as an excellent thermoelectric n-type material and studied extensively [5,6,7]. Isoelectronic substitution of the X element has led to several reported ZT values in the range of 1 [8,9,10,11,12,13,14]. 

However, reports on the thermoelectric performance of modules based on half-Heusler (HH) compounds still remain scarce. Poon *et al.* [15] reported a remarkable performance of a single n- and p-type leg device with a maximum power output of 423 mW at a temperature gradient of 704 K. However, dimensions of the legs and details for the contacting material are not given, which makes an evaluation of these results difficult. Furthermore, several publications reported thermoelectric modules, which are made of full Heusler compounds with large numbers of legs [16,17], reaching power densities of up to 0.2 W/cm^2^.

In this work, we report the synthesis and characterization of thermoelectric Ti_0.33_Zr_0.33_Hf_0.33_NiSn compounds, which were afterwards used for the construction of a unileg module containing only n-type legs. Subsequently, the module was characterized in a homemade module measurement test stand. 

## 2. Experimental

Half-Heusler compounds with the nominal composition Ti_0.33_Zr_0.33_Hf_0.33_NiSn were prepared by arc melting of the corresponding amounts of the metals under a purified argon (6.0) atmosphere. To ensure a good homogeneity, the samples were remelted and turned several times. Additionally, they were subjected to a 2-week heat treatment in quartz glass ampoules under argon atmosphere at 1173 K, followed by quenching in ice water. The electrical resistivity, ρ, and the Seebeck coefficient, S, were investigated using a RZ2001i Ozawa Science measurement system (Ozawa Science Co., Nagoya, Japan). The thermal conductivity, κ, was evaluated indirectly by measurements of the thermal diffusivity, α (Netzsch LFA 457 Microflash, Netzsch, Selb, Germany), and the specific heat, c_p_ (Netzsch DSC 404 C, Netzsch, Selb, Germany). The density was determined by Archimedes’ method. The unileg module, consisting of 4 n-type legs with a length of 4 mm and cross sections of 2 mm × 2 mm, was characterized using a homemade test stand. This thermoelectric generator (TEG) measurement system consists of an electrical heater with a Proportional-Integral-Derivative (PID) temperature controller, a cooling device (NESLAB RTE-110), a temperature logger and an electronic load (H&H, ZS 506-4). Subsequently, for selected temperatures of the hot and the cold side, the power output of the module was measured by applying different loads.

## 3. Results

**Figure 1:**
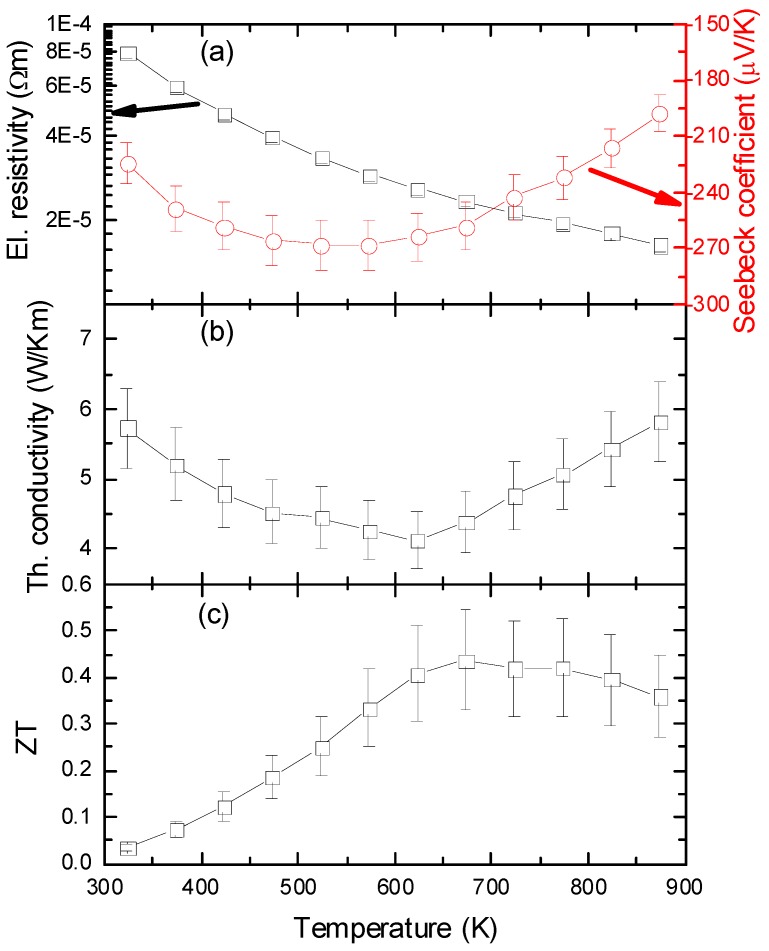
(**a**) Left ordinate electrical resistivity, ρ, plotted against temperature; right ordinate Seebeck coefficient, S, against temperature; (**b**) thermal conductivity, κ, plotted against temperature; (**c**) dimensionless Figure of Merit ZT in the temperature range from 300 K to 900 K.

The measurements of the thermoelectric properties in the temperature range from 300 K to 900 K are presented in Figure 1. The electrical resistivity is decreasing over the whole temperature range, indicating semiconducting behavior and reaching a minimum of 18 μΩ·m at 870 K. The Seebeck coefficient is negative in the whole studied temperature range, indicating n-type conductivity. The absolute value of S is increasing in the temperature range from 300 K to 600 K to a maximum value of S = −270 μV/K, followed by a decrease to S = −190 μV/K. The thermal conductivity is rather high, starting from 5.7 W/mK at room temperature and decreasing to a minimum of 4.1 W/mK at 620 K, after which it increases again to a value comparable to the one at room temperature, at 870 K. A decreasing κ confirms a predominant contribution from phonon–phonon scattering, whereas the increase in κ at high temperatures is due to the enhanced contribution of electronic thermal conductivity. Furthermore, the minimum of lattice thermal conductivity (κ_l_) and total thermal conductivity (κ) are close to the temperature where the Seebeck coefficient peaks, a signature of bipolar thermal conduction. Subsequently, in Figure 1c, the calculated ZT value is presented, showing a maximum ZT value of 0.44 around 675 K. At even higher temperatures, ZT decreases, due to a decreasing Seebeck coefficient and an increasing thermal conductivity.

Furthermore, a part of the samples was used for assembling a thermoelectric generator (TEG) based on half-Heusler alloys. Traditional thermoelectric generators consist of both n- and p-conducting legs. In the absence of a good p-type half-Heusler material, it was decided to use only the n-type semiconducting (TiZrHf)NiSn, as described above. This approach was used by Lemmonier *et al.* [18] using only n-type Ca_0.95_Sm_0.05_MnO_3_ legs for a TEG. 

**Figure 2:**
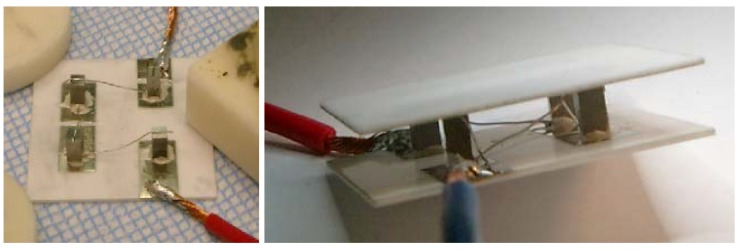
Photographs of the assembled module.

In the unileg device, platinum strips connecting the bottom of one leg with the top of the adjacent leg serve as electrical connections replacing the missing p-type legs. The main disadvantage of this unileg approach is that packing densities are low, thus limiting the market acceptance. However, in the case of our test modules, a simplified manufacturing process compensates for this drawback. In Figure 2, photographs of the assembly of the module and the finished module are shown.

The (TiZrHf)NiSn legs are sandwiched between two Al_2_O_3_ layers in order to ensure mechanical stability and a good thermal contact to the temperature control media. The conducting paths were screen printed onto the aluminum-oxide plates using gold- and silver-based pastes (DuPont), followed by a heat treatment at 1273 K to burn the coatings in. Subsequently, the HH-legs were glued to the Al_2_O_3_ layers with the same silver paste, and platinum stripes serve as electrical connections, substituting the p-type legs. 

The performance of the unileg module was characterized using a homemade TEG measurement system. The electrical power output of the module was measured at different preset temperature gradients. The hot side of the module was heated up to 868 K, while the cold side was water cooled and kept at 303 K. For the sake of reproducibility, the temperature gradients were measured outside of the Al_2_O_3_ plates with a thermocouple of type K, ensuring a good thermal contact. The thermal gradient along the thermoelectric legs will be much smaller, due to the thermal resistances of these plates.

**Figure 3:**
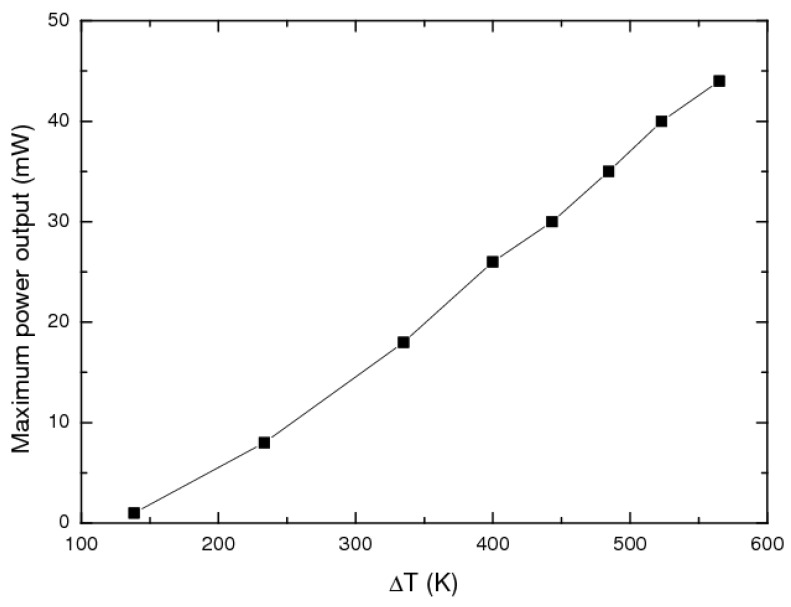
Maximum power output of the half Heusler based unileg module as a function of the temperature gradient.

Figure 3 shows the maximum power output at different temperature gradients from 130 K to 565 K.

The power output is a nearly linear function of the temperature difference, reaching a value of 44 mW at Δ*T* = 565 K. This corresponds to power densities of 275 mW/cm^2^ or 700 mW/cm^3^. It has to be noted that these results were achieved without optimization of the contact resistance. In oxides, for instance, Funahashi *et al.* could improve the power output by nearly a factor of three by improving the contacting material by using a silver/metal oxide compound [19].

In order to evaluate these results, Table 1 summarizes materials, maximum power output at a given temperature gradient, dimensions and the number of legs of several thermoelectric generators based on Heusler or half-Heusler compounds. For comparison, also the power density of each module is specified, where possible.

**Table 1:** Comparison of different modules based on Heusler and half-Heusler compounds.

Module	Material	Δ*T* (K)	P_out, Max_ (mW)	Leg dimensions (mm)	Power density	
					(mW/cm^2^)	(mW/cm^3^)
HH-Empa (this work)	Ti_0.33_Zr_0.33_Hf_0.33_NiSn unileg	565	44	4-times 2 × 2 × 4	275	700
Heusler1 Mikami [16]	Fe_2_VAl_0.9_Si_0.1_/Fe_2_V_0.9_Ti_0.1_Al	280	940	36-times diameter 5 × 5	133	266
Heusler2 Mikami [17]	Fe_2_V_0.84_Ti_0.16_Al_0.97_Sb_0.03_/Fe_2_VAl_0.9_Si_0.07_Sb_0.03_	280	2500	36-times 4.5 × 4.5 × 4.2	342	816
HH Poon [15]	Hf_0.3_Zr_0.7_CoSn_0.3_Sb_0.7_/Hf_0.6_Zr_0.4_NiSn_0.995_Sb_0.005_	704	423	2 legs	–	–
Funahashi [19]	Ca_2.7_Bi_0.3_Co_4_O_9_/La_0.9_Bi_0.1_NiO_3_	500	94	2-times 3.7 × 4.5	105 (282)	246 (660)
Tomes [20]	La_1.98_Sr_0.02_CuO_4_/CaMn_0.98_Nb_0.02_O_3_	622	88	4-times 4.5 × 4.5	108	216

The TEG presented in this work delivers power densities comparable to the second generation full Heusler modules of Mikami *et al*. [17]. Regarding the temperature gradient, it has to be noted that in [17], the hot side temperature was calculated from the open circuit voltage and not measured. Due to missing values for the leg dimensions, the promising results of Poon *et al.* [15] cannot be evaluated in terms of power density. Furthermore, two examples of thermoelectric converters for high temperature application based on oxides can be found for comparison. In the case of a non-optimized conducting material in [19], as well as in the work of Tomes *et al.* [20] from our group, the power density of the module is nearly three-times smaller than the one obtained in this work. This is due to the much higher mean ZT value of the material used in this work. Our results demonstrate the huge potential of TEGs based on half-Heusler alloys, as an even better performance can be expected with an optimized contact material. A detailed study is planned in a next step. Furthermore, the use of materials with recently published much higher ZT values [10,11,12,13,14] will even further increase the performance of TEG based on half Heusler compounds. 

## 4. Summary and Conclusion 

Ti_0.33_Zr_0.33_Hf_0.33_NiSn half-Heusler compounds were prepared by arc melting, and the thermoelectric characterization up to 900 K yielded a maximum ZT = 0.44 around 675 K. Consequently, from the material, a unileg module was produced and characterized using a homemade high temperature module measurement test stand. The maximum power output was 44 mW at Δ*T* = 565 K, which corresponds to 275 mW/cm^2^ and a volumetric power density of 700 mW/cm^3^. Comparison to the literature showed that only very little and incomplete data is at hand reporting on TEG based on half-Heusler compounds. Where a comparison of the power densities could be drawn, our results were in the range of the best ones published. 

Due to the relatively low ZT values of the used composition and the not yet optimized contact resistance, further improvements are to be expected. 
